# Characterization of a dominant mutation for the liguleless trait: *Aegilops tauschii* liguleless (*Lg*^*t*^)

**DOI:** 10.1186/s12870-019-1635-z

**Published:** 2019-02-15

**Authors:** Alina E. Dresvyannikova, Nobuyoshi Watanabe, Alexander F. Muterko, Alexander A. Krasnikov, Nikolay P. Goncharov, Oxana B. Dobrovolskaya

**Affiliations:** 1 Institute of Cytology and Genetics, SB RAS, Lavrenvieva ave. 10, Novosibirsk, 630090 Russia; 2 Novosibirsk State University, Pirogova, 2, Novosibirsk, 630090 Russia; 3 College of Agriculture, Ibaraki University, Ibaraki, 300-0393 Japan; 4 Central Siberian Botanical Garden SB RAS, Zolotodolinskaya Str., 101, Novosibirsk, 630090 Russia

**Keywords:** Leaf development, Liguleless mutant, *Aegilops tauschii*, Diversity arrays technology, DArT

## Abstract

**Background:**

Leaves of Poaceae have a unique morphological feature: they consist of a proximal sheath and a distal blade separated by a ligular region. The sheath provides structural support and protects young developing leaves, whereas the main function of the blade is photosynthesis. The auricles allow the blade to tilt back for optimal photosynthesis and determine the angle of a leaf, whereas the ligule protects the stem from the entry of water, microorganisms, and pests. Liguleless variants have an upright leaf blade that wraps around the culm. Research on liguleless mutants of maize and other cereals has led to identification of genes that are involved in leaf patterning and differentiation.

**Results:**

We characterized an induced liguleless mutant (LM) of *Aegilops tauschii* Coss., a donor of genome D of bread wheat *Triticum aestivum* L.. The liguleless phenotype of LM is under dominant monogenic control (*Lg*^*t*^). To determine precise position of *Lg*^*t*^ on the *Ae. tauschii* genetic map, highly saturated genetic maps were constructed containing 887 single-nucleotide polymorphism (SNP) markers derived via diversity arrays technology (DArT)seq. The *Lg*^*t*^ gene was mapped to chromosome 5DS. Taking into account coordinates of the SNP markers, flanking *Lg*^*t*^, on the pseudomolecule 5D, a chromosomal region that contains this gene was determined, and a list of candidate genes was identified. Morphological features of the LM phenotype suggest that *Lg*^*t*^ participates in the control of leaf development, mainly, in leaf proximal–distal patterning, and its dominant mutation causes abnormal ligular region but does not affect reproductive development.

**Conclusions:**

Here we report characterization of a liguleless *Ae. tauschii* mutant, whose phenotype is under control of a dominant mutation of *Lg*^*t*^. The dominant mode of inheritance of the liguleless trait in a Triticeae species is reported for the first time. The position of the *Lg*^*t*^ locus on chromosome 5DS allowed us to identify a list of candidate genes. This list does not contain *Ae. tauschii* orthologs of any well-characterized cereal genes whose mutations cause liguleless phenotypes. Thus, the characterized *Lg*^*t*^ mutant represents a new model for further investigation of plant leaf patterning and differentiation.

**Electronic supplementary material:**

The online version of this article (10.1186/s12870-019-1635-z) contains supplementary material, which is available to authorized users.

## Background

**Fig. 1:**
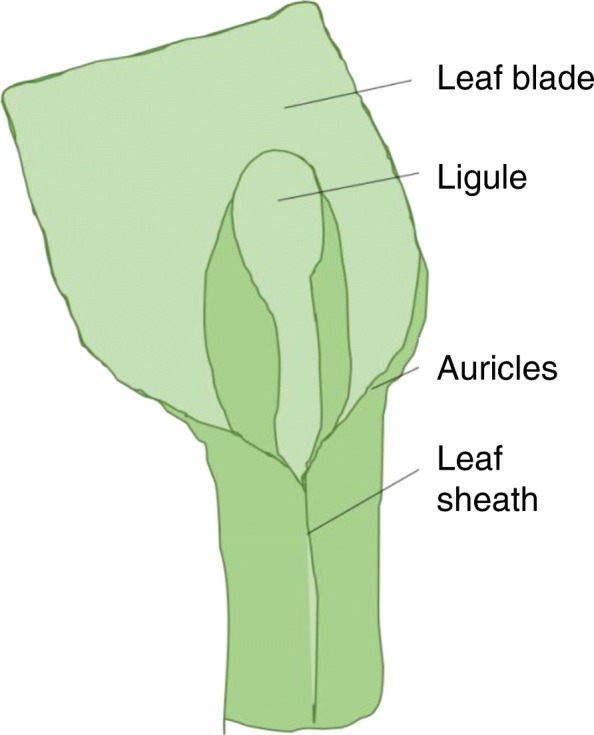
Leaf structure of Triticeae

The leaf of the family Poaceae has a characteristic feature: its distal and proximal parts are separated by a ligular region consisting of a ligule and a pair of auricles (Fig. 1). The ligule is well developed in the majority of subfamily Pooideae representatives, but most of genus Echinochloa species have no ligule. The thickness of a ligule varies from leathery to pleat. The ligule is considered a progressive organ that prevents penetration of water, dust, and microorganisms into the leaf sheath [1].

Formation of the distal–proximal axis of differentiation and development of the organs of the ligular region occurs at the early stages of leaf development. A disturbance of these processes leads to incomplete development or the absence of a ligule and auricles [2]. Analysis of maize (*Zea mays* L.) mutants with abnormal ligular region development has enabled characterization of ligular-region development and identification of genes that control this process [2, 3].

The most fully characterized gene is *Z. mays* gene *LIGULELESS 1* (*LG1*), encoding the transcription factor SQUAMOSA PROMOTER BINDING PROTEIN (SBP), which is necessary for the development of the verge of the leaf blade and leaf sheath. It is assumed that the protein LG1 is localized in the cell nucleus [4]. Recessive mutant *lg1* does not have a ligule and auricles, and the leaves are narrower and positioned vertically (upright) [5]. The ligule is absent on the first 10 leaves; however, a rudimentary ligule without the auricles develops on subsequent leaves [6]. All characterized mutant genes have shown a recessive mode of inheritance.

Gene *LIGULELESS 2* (*LG2*) of maize also controls the development of the ligule. *LG2* encodes a basic-leucine zipper transcription factor. The *lg2* recessive mutant does not form a normal preligular region [7]. The first and sometimes the second leaf have no completely developed ligule or auricles, but these organs develop on the margins of the third leaf. On all subsequent leaves, the ligule is more developed, whereas on the last leaves it is normal. It is assumed that the *lg2* gene determines localization of the ligule and auricles, and the *lg1* gene controls their development [8, 9].

Gene *KN1* (*KNOTTED1*) of maize regulates the expression of key specific transcription factors in leaves. *KNOX* and the *LIGULELESS 3* gene (*lg3*) are normally expressed in a maize sheath, and their products bind to *KN1*. In lines with a knockout of this gene, the leaf is normal [10]. Maize gene *knotted1* (*kn1*) is the first homeobox gene identified in plants. Genes of the *kn1* family have been isolated in many other plant species and are subdivided into two classes. The first class includes genes *kn1* and *rough sheath1* of maize, *OSH1* and *OSH15* of rice*, HvKnox3* of barley, and *STM1* and *KNAT1* of *Arabidopsis thaliana* (L.) Heynh. These genes are mainly expressed in the tissues of the meristem and affect development of the plant [11].

The functions of the *Lg3* gene are less clear. Genes paralogues *lg4a* (*knox 11*) and *lg4b* (*knox 5*) encode proteins with degenerate functions. Genes *Lg3* and *Lg4a* (and *-b)* in maize are found on chromosomes 3S and 8 L, respectively [12]. In rice, orthologs *OSH6* and *OSH71* are located on chromosome maps in areas of conservative synteny.

The maize *Liguleless narrow* (*Lgn*) semidominant mutation determines the pattern of leaf development. The mutant plant leaves are narrow, with an irregularly developed ligule and auricles, and inflorescences are less branching. In homozygotes for this gene, reproductive structures are not formed.

The genes that regulate the distal–proximal leaf differentiation and formation of the ligular region of Triticeae have been less studied. Unique plants of *Triticum durum* Desf. that lack a ligule and auricles were found in Cyprus [13].

Liguleless forms of rye were found in the Pamir region in 1918 [14]. In liguleless plants, the ligule is absent only in the first leaves, but in the leaves above, the ligule has been observed. Genetic analysis showed that the ligulelessness in rye is determined by a recessive allele of one gene. The gene was named *el* (*eligulatum*): a leaf without a ligule [15]. The gene was mapped to chromosome 2RL [16].

Mutant plants of barley *Hordeum vulgare* L. have no ligule and auricles on all leaves, and the disruption of formation occurs in the early stages of development. The gene that determines the ligulelessness phenotype of the leaf was localized on the long arm of chromosome 2H, a candidate gene for this morphological gene was identified; it is *hvLG1*, an ortholog of the *LG1* gene of maize [17].

Wheat *Triticum* L. has variants with a liguleless leaf phenotype; they lack a ligule and auricles on all leaves. Three morphological genes have been identified whose recessive alleles determine the liguleless phenotype: *lg1*, *lg2* and *lg3*. These genes were localized on molecular-genetic maps of chromosomes 2BL, 2DL, and 2AL, respectively [18–23].

An induced mutant with a liguleless phenotype was obtained in a wild representative of the tribe Triticeae—*Aegilops tauschii* Coss (2*n* = 14, DD), which is a diploid donor of genome D of hexaploid wheats [24]. Although in *T. aestivum* [18], *T. durum* [25], and *T. monococcum* L. [19], the absence of a ligule is a recessive trait, in the *Ae. tauschii* mutant, the absence of the ligule is dominantly inherited. It was found that the gene determining the mutant phenotype of *Lg*^*t*^ (*Liguleless Ae. tauschii*) is linked to a microsatellite marker of chromosome 2DL: *Xbarc159* (9.3 cM); however, *Xbarc159* was not linked to any other 2DL markers [26].

Here we characterized the *Ae. tauschii* liguleless mutant by light microscopy and scanning electron microscopy (SEM), and according to the features of the mutant phenotype, we proposed a function of its causative gene in leaf development. Using a diversity arrays technology (DarT)seq approach, genetic maps of seven *Ae. tauschii* chromosomes were constructed, and the gene determining the *Ae. tauschii* liguleless phenotype was genetically mapped. The position of the gene on a molecular-genetic map became a basis for identification of a list of candidate genes.

## Methods

### Plant material

The *Ae. tauschii* Liguleless Mutant was artificially produced by Dr. T. Makino, Agricultural Research Center, Tsukuba, Japan [26]. A near-isogenic line (NIL) Liguleless-G3489 was created from the cross of G3489 (recurrent parent) with the Liguleless Mutant by backcrossing to G3489. After six backcrosses, heterozygous BC6F1 plants were selfed, and plants with the dominant liguleless and recessive ligule phenotypes were selected. After further selfing of the selected liguleless and ligule plants, the Liguleless-G3489 near isogenic line (NIL-*Lg*^*t*^ throughout this paper) and its sibling line (SIB-line throughout this paper) were obtained. *Ae. tauschii* accession G3489 with a ligule phenotype was kindly provided by Prof. J. G. Waines, the University of California at Riverside.

To map the *Lg*^*t*^ gene (Liguleless in *Ae. tauschii*), an F_2_ mapping population derived from a cross between the Liguleless Mutant and KU2126 was developed. *Ae. tauschii* accession KU2126 with a ligule phenotype was collected by Kihara et al. (1965) [27]. F_2_ and the parental plants were grown under greenhouse conditions. Leaf phenotypes of F_2_ plants were estimated approximately 14 and 30 days after sowing.

### Light microscopy and SEM analysis

Leaf morphology of KU2126, G3489, and of the Liguleless Mutant was examined using a Carl Zeiss Stereo Discovery V12 light microscope (Carl Zeiss Microscopy GmbH, Germany). The images were captured with the AxioCam MRc-5 (Carl Zeiss Microscopy GmbH, Germany). Morphological features of ligule regions were examined under a scanning electron microscope, SEM (TM-1000, Hitachi Co., Ltd., Japan). Dissected leaf segments were examined in low vacuum (30–50 Pa) at an accelerating voltage of 15 kV.

### DNA isolation and genotyping

Total genomic DNA was isolated from leaf material of individual F_2_ plants, NIL-*Lg*^*t*^, SIB-line, and parental lines according to the method of Plaschke et al. (1995) [28]. Next, 20 μl of a 70 ng/μl DNA solution of each sample (F_2_ plants, Liguleless Mutant, and KU2126) was sent to Diversity Arrays Technology (DArT) Pty Ltd., Australia (http://www.diversityarrays.com) for a whole-genome scan by the DArTseq method. Whole-genome genotyping of the 92 F_2_ plants and 2 parental lines was carried out following the protocol described by Akbari et al. [29] by the wheat DArTseq assay (wheat GBS 1.0). Raw sequence data (single-nucleotide polymorphism [SNP] scoring data) of each clone are given in Table S1. DArTseq-derived SNP markers were scored as follows: 0 = reference allele homozygote, 1 = SNP allele homozygote, 2 = heterozygote, and “-” = double null/null allele homozygote (the absence of the fragment with the SNP in genomic representation). Although DArTseq generates two types of data, silicoDArTs and SNPs, here we present DArTseq-derived SNP data as a codominant highly informative kind of markers.

### Linkage map construction

Genotype data were uploaded to the MultiPoint Ultra-dense (ULD) mapping program (MultiQTL Ltd., Haifa, Israel) and processed as an F_2_ population [30]. Markers with more than 15% of missing data and markers with large segregation distortion (*χ*^2^ > 21) were removed. Multilocus ordering was determined via an algorithm based on the evolutionary optimization strategy [31, 32] with maximum likelihood estimation to calculate pairwise recombination fractions (*rf*) for all marker pairs. Preliminary clustering and assignment of markers to a linkage group (LG) were evaluated at an *rf* = 0.15 threshold. Stability of marker neighborhoods within a LG was evaluated by jackknife resampling (10 jackknife resampling runs), with repeated verification of marker order and removal of unreliable markers. Single markers were added to the map using the “extending linkage group” function with the coefficient of enlargement increased stepwise from 1.0 to 1.4. Markers mapping to the same location were grouped and represented by a single delegate: a skeleton marker. Stable LGs were joined terminally by incrementally increasing the recombination threshold, with a final *rf* of 0.30. The distances (in centimorgans, cM) were calculated using the Kosambi mapping function. Maps were drawn by means of MapChart 2.2 (http://www.biometris.nl/).

To determine the positions of mapped DArTseq-derived SNPs on *Ae. tauschii* pseudomolecules, we BLASTed SNP-containing sequences linked to the gene of interest (*Lg*^*t*^) against the genome sequence of *Ae. tauschii* (http://aegilops.wheat.ucdavis.edu/ATGSP/blast.php) with e-value < 1e-10. Based on the obtained sequences, primer pairs were designed (https://www.genscript.com/tools/pcr-primers-designer) to amplify SNP-containing regions (up to 300 bp; Additional file 1: Table S4) in NIL-*Lg*^*t*^, SIB-line, Liguleless Mutant, and G3489. Sequences of SNP-containing amplicons were obtained by direct Sanger sequencing of PCR products using the BigDye Terminator v3.1 cycle sequencing kit (Applied Biosystems). Fluorescently terminated extension products were separated on a capillary ABI 3130xl Genetic Analyzer (Applied Biosystems). Alleles of SNP markers linked to the *Lg*^*t*^ gene were thus identified in NIL-*Lg*^*t*^ and SIB-line.

SSR (simple sequence repeats) markers with a known position on *Ae. tauschii* pseudomolecule 5D [33, 34] were also used to genotype NIL-*Lg*^*t*^ and SIB-line and to determine the segment(s) of introgression. SSR-analysis was performed as previously described [35] on ABI 3130xl. All SSR primers used in this study are listed in Additional file 2: Table S6. Information on the SSR markers employed here is available at http://www.graingenes.org.

The *Lg*^*t*^ gene was mapped as a single gene in JoinMap 6 software (https://kyazma.nl) and as a quantitatively inherited trait. The position of the quantitative trait locus (QTL) for the liguleless trait (*qLg*^*t*^) was identified by interval mapping in the MultiQTL software [36]. To determine QTL threshold limit of detection (LOD) value, 200 permutations were carried out using option “Comparing Hypotheses H1 → H0.” To estimate standard deviations of the main parameters, bootstrap analysis was applied.

#### In silico gene recognition and annotation

The gene recognition was carried out in two ways, using a part of DNA sequence of reference genome and using a set of *ab initio* predicted gene models. The region of interest was cut from the pseudomolecule of chromosome 5D of *Ae. tauschii* (ROI-5D), retrieved from the GenBank (BioProject PRJNA341983). *Ab initio* predicted coding sequences (CDS) from ROI-5D were downloaded from the ATGSP repository (http://aegilops.wheat.ucdavis.edu/ATGSP/annotation/). BLAST was carried out for ROI-5D and *ab initio* predicted CDS against plant CDS databases (ENA Coding Sequence Plant Release 130, ftp://ftp.ebi.ac.uk/pub/databases/ena/coding/release/std/) using BLASTN 2.8.0+ [37]. Local alignments were assembled and clustered. Assemblies with identity or coverage of reference sequence less then 80% were filtered out. Gene ontology was annotated with QuickGO and GOA using REST API (https://www.ebi.ac.uk/QuickGO/api/index.html). The non-redundant set of proteins was obtained for each cluster. Genes, annotated as mobile genetic elements were filtered out.

## Results

### Phenotypic characterization of the *Liguleless Mutant* of *Ae. tauschii*

**Fig. 2:**
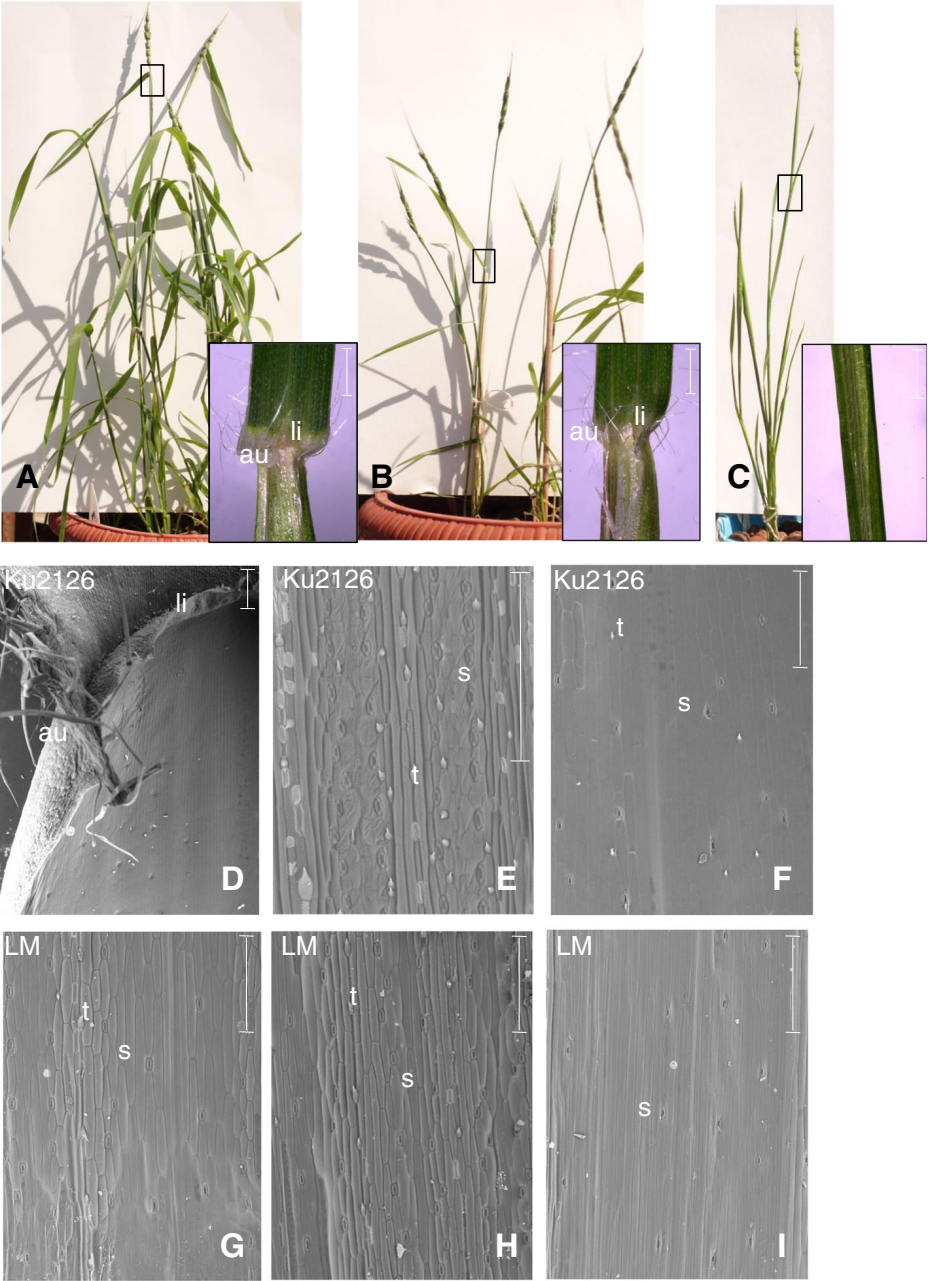
**a**–**c** Characteristics of the *Ae. tauschii* liguleless mutant and of normal adult plants. **a** G3489, (**b**) Ku2126, and (**c**) Liguleless Mutant (LM). Light microscopy images of the ligular region of each line are at the bottom. Scale bars: 1 mm. **d**–**i** Scanning electron microscopy images of Ku2126 (wt) and Liguleless Mutant (LM) leaves. **d** and **g** The ligule region of wild-type line Ku2126 (**d**) and of LM (**g**). **e** and **h** The leaf blade of Ku2126 (**e**) and LM (**h**). **f** and **i** Leaf sheaths of Ku2126 (**f**) and LM (**i**). Scale bars: 300 nm; au, auricle; li, ligule; t, trichomes; s, stomata

The Liguleless Mutant (LM), in contrast to nonmutant G3489 and KU2126 lines, has upright leaves (Fig. 2a–c). Light microscopy and SEM showed differences in the structure of their ligular regions. The ligule and auricles were completely absent in LM, in contrast to G3489 and KU2126, which have leaves of the wild-type phenotype (Fig. 2). Neither leaf blade nor sheath morphology were affected in LM (Fig. 2h, i), but the lengths of leaf blades were shorter in LM and NIL-*Lg*^*t*^ compared to the SIB-line. The number of liguleless leaves did not affected by the environment, and the trait manifestation throughout ontogenesis did not change.

Reproductive development of LM was normal; it produced inflorescences (spikes) of normal morphology with fertile florets, but spike length was slightly but significantly (*t* = 1.77; *P =* 0.1) shorter in NIL-*Lg*^*t*^ (4.9 ± 0.8 cm) compared to the SIB-line (7.3 ± 1.0 cm). The number of spikelets per spike in NIL-*Lg*^*t*^ (9) was slightly less than that in its SIB-line (14), but the difference was not significant, and the spike densities were not different between these two lines.

We did not observe a significant difference in the tiller number between NIL-*Lg*^*t*^ and SIB-line. Thus, the mutation causes an alteration in the ligular region morphology and does not affect reproductive development and axial bud formation (tillering). The LM phenotype is suggestive of the role of its causative gene in ligular-region development. Probably, gene *Lg*^*t*^ pleiotropically affects leaf growth processes, and its dominant mutation reduces leaf blade size (primarily leaf blade length).

### Genetic characterization of the LM

F_1_ hybrids from the LM/KU2126 cross had a liguleless leaf phenotype. The F_2_ progeny showed either liguleless or ligule phenotypes. The segregation ratio was consistent with three ligule (36 plants) to one liguleless (77 plants) phenotypes, confirming the monogenic dominant inheritance of the trait (*χ*^*2*^ = 2.8, *P* < 0.05) reported by Amagai et al. [26]. The dominant mode of the liguleless trait inheritance is different from the results of all other studies on liguleless phenotypes in genus *Triticum*. In hexaploid wheats, the liguleless phenotype results from the absence of dominant ligule-conferring alleles in homeologous group 2 chromosomes: usually *lg1* on chromosome 2B and *lg2* on chromosome 2D [18]. The *Ae. tauschii* gene for the liguleless trait (*Liguleless* in *Ae. tauschii*, *Lg*^*t*^) was transferred to a wheat hexaploid synthetic line, SynW-1; and it was found that dominant gene *Lg*^*t*^ conferred a liguleless phenotype at the hexaploid level [26].

The *Lg*^*t*^ gene, determining the liguleless phenotype of LM, was shown to be linked to the *Xbarc159* marker of chromosome 2DL, and those authors concluded that *Lg*^*t*^ is located on chromosome 2DL [26]. Nonetheless, the *Xbarc159* locus was not linked to the other 2D microsatellite loci [26], but on wheat chromosome 2D, *Xbarc159* and *Xgwm301* are closely linked (~ 5 cM) (https://wheat.pw.usda.gov/GG3/). Moreover, the *Lg*^*t*^ (2DL) and *Iw2* (2DS) genes were found to be inherited independently in the same mapping population [26].

### Construction of *Ae. tauschii* genetic maps

To determine the position of the *Lg*^*t*^ locus on a *Ae. tauschii* chromosome, the DArTseq approach, which allows for construction of highly saturated molecular-genetic maps, was applied in the current study. A total of 3934 polymorphic DArTseq-derived SNP markers were used in this study (Additional file 3: Table S1). Of these, 3409 DarT-seq derived SNPs (605 skeleton markers) were mapped to 25 linkage groups (LG). After removing unreliable markers and merging linkage groups, we obtained seven LGs. A total of 887 markers were mapped. The constructed genetic maps spanned 1087.89 cM, with individual chromosomes ranging from 113.68 cM (3D) to 191.15 cM (2D) (Additional file 4: Table S2). The average distance was 3.54 cM. The number of skeleton markers on the different chromosomes ranged from 29 (2D and 6D) to 68 (5B), and these markers were evenly distributed on each chromosome. A total of 68 gaps from 5 to 25 cM were observed, and the largest gap, 22.93 cM, was found on chromosome 4D (Additional file 4: Table S2). The constructed maps of all seven linkage groups of *Ae. tauschii* containing skeleton markers are presented in Additional file 5: Figure S1, and detailed information about positions of all the mapped markers is given in Additional file 6: Table S3.

### Mapping the *Lg*^*t*^ gene

The *Lg*^*t*^ gene was previously assigned to chromosome 2D by an SSR assay [26]. To compare SSR- and SNP-derived chromosome 2D linkage maps, we employed *Xbarc159* (SSR marker linked to *Lg*^*t*^*)* and *Xgwm301* (most distally located marker of 2DL [26]) to integrate them into the SNP linkage map constructed in the current study. We genotyped the F_2_ plants from the LM/KU2126 cross, using the GWM301 microsatellite marker, and integrated *Xgwm301* into the 2D SNP map. The *Xgwm301* locus was found to be linked to 21311252_0026 (0.6 cM, distally) and to 1193685_0018 (0.6 cM, proximally): the SNP markers of the 2DL distal region (Additional file 7: Figure S2). These mapping results are consistent with those of Amagai et al. [26]. BARC159 failed to be amplified in *Ae. tauschii* LM, KU2126, and 16 F_2_ plants from the LM/KU2126 cross, but the product of expected size (220 bp) was obtained in a *T. aestivum* cv. Chinese Spring DNA sample; the latter served as a positive control. Thus, our results could not confirm the location of the *Lg*^*t*^ gene locus on 2DL because we did not observe amplification of BARC159, which was previously shown to be linked with *Lg*^*t*^.

**Fig. 3:**
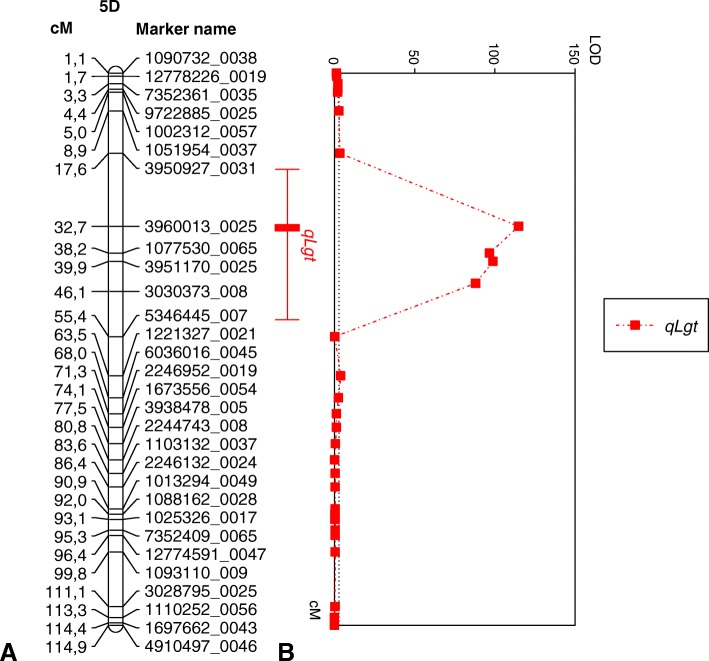
**a** The genetic linkage map of *qLg*^*t*^*.* Genetic distances are indicated on the left side of linkage group in centimorgans (cM), the marker names are shown on the right. **b** The results of the QTL mapping. In the chart, the red curve indicates the LOD value, according to the MapChart software

Given that the liguleless phenotype of LM is under monogenic control, first, we mapped *Lg*^*t*^ as a single gene. The gene was mapped to chromosome 5DS, distally to the 3960013_0025 marker (11.4 cM) and proximally to the 1077530_0065 marker (8.8 cM) (Additional file 8: Figure S3). The *Lg*^*t*^ mapping caused extension of the 3960013_002 –1077530_0065 interval from 5.5 cM to 22.2 cM. This extension might be a result of miss-scoring of F_2_ plants’ phenotypes (phenotyping errors), or it was a consequence of incomplete penetrance of the *Lg*^*t*^ mutation or resulted from the influence of a minor gene(s), which modifies manifestation of the *Lg*^*t*^ mutation. To avoid any mapping errors and to identify possible QTL(s) with a minor effect, we mapped the *Lg*^*t*^ gene as a quantitatively inherited trait (QTL). A unique QTL, *qLg*^*t*^, (LOD = 114.85, *P* = 0.0025) was identified on chromosome 5DS (Fig. 3), which was completely colocalized with the *Lg*^*t*^ position on 5DS (Additional file 8: Figure S3). We did not identify any more QTLs, including those with minor effects, on any *Ae. tauschii* chromosomes.

Localization of a gene for the liguleless trait on a 5-group chromosome of Triticeae species is shown for the first time. To confirm the mapping results, we determined a segment of introgression in NIL-*Lg*^*t*^ and its SIB-line by comparing 5DS chromosomes of NIL*-Lg*^*t*^ and the SIB-line by means of genotyping with SNP and SSR markers. The donor of the liguleless trait of NIL-*Lg*^*t*^ was LM, but the recurrent parent, G3489, was different from that used for mapping population development (Ku2126); thus, allelic composition of SNP loci was unknown in G3489. To identify alleles of SNP markers linked to *Lg*^*t*^, we designed primers for amplification of the SNP-containing regions. In total, 10 primer pairs were designed and used for amplification of SNP-containing regions (Additional file 1: Table S4). Sequences of SNP-containing regions (300 bp) in LM, Ku2126, G3489, NIL-*Lg*^*t*^, and SIB-line were obtained by direct Sanger sequencing (Additional file 9: Table S5). SNP allele composition that was found in LM and Ku2126 confirmed the composition determined by DArTseq (Additional file 1: Table S4), but most of the SNPs (8 of 10) were nonpolymorphic between the donor of the liguleless trait (LM) and the recurrent parent (G3489). Nevertheless, we found three additional SNPs in two sequences containing markers 3960013_0025 and 1202478_0022 (Additional file 9: Table S5). Thus, four polymorphic SNP markers were chosen to characterize 5DS of NIL-*Lg*^*t*^ and the SIB-line.

**Fig. 4:**
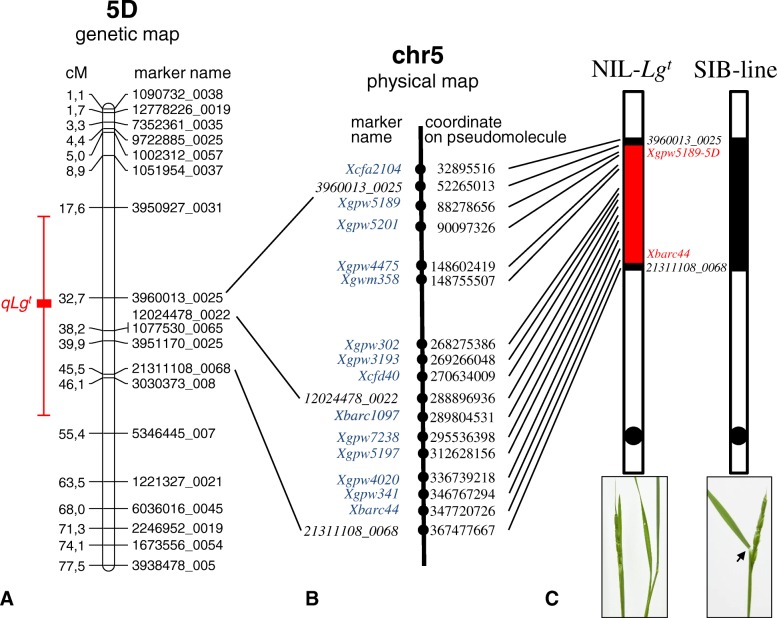
**a** The genetic map of chromosome 5D, distances are shown in centimorgans (cM). **b** A physical map of chromosome 5D, the position of markers is shown on the right. **c** Graphical representation of chromosome 5D of NIL-*Lg*^*t*^ (of the left) and SIB-line (on the right), the red square indicates introgression from the Liguleless Mutant, the dots denote a centromere, and the arrow marks a ligule

In addition to SNP markers, we used SSRs that were previously assigned to *Ae. tauschii* chromosome 5D, and their coordinates on pseudomolecule chr5 were known [33, 34]. A total of 15 SSR markers were used; of these, 14 were polymorphic and applied to characterization of chromosome 5DS in NIL-*Lg*^*t*^ and the SIB-line (Additional file 2: Table S6). Taken together, the results of genotyping with SNP and SSR markers showed that the 5DS chromosome of NIL-*Lg*^*t*^ possesses a segment of introgression from the liguleless trait donor (LM), whereas the same 5DS region in the SIB-line does not have any introgression (Fig. 4). These results confirmed the obtained mapping results. Thus, the *Lg*^*t*^ gene is located on chromosome 5DS of *Ae. tauschii.*

Taking into account the coordinates of SNP markers on pseudomolecules 5D (3960013_002 and 1077530_0065), which flanked the *Lg*^*t*^ gene (Fig. 3), a chromosomal region that contains this gene was determined. Gene ontology (GO) within ROI-5D in terms of a molecular function, biological process, and cellular component was used for annotation. Among the 87 *ab initio*–predicted genes located on ROI-5D (the 3960013_002 and 1077530_0065 interval), only 60 were annotated, in contrast to 183 out of the 187 genes identified by similarity searching in the CDS database (Additional file 10: Table S7). Among the 183 genes, the molecular function was predicted for 89, while a biological process and cellular component for 68 and 96 genes, respectively. Forty-four genes were annotated in all three GO categories. Among 35 unique biological processes the “ATP synthesis coupled proton transport” (GO:0015986, 15 proteins), “photosynthesis” (GO:0015979, 13 proteins), and “regulation of transcription, DNA-templated” (GO:0006355, 11 proteins) were dominant. Predominant molecular functions were “DNA binding” (GO:0003677), “protein heterodimerization activity” (GO:0046982), and “proton transmembrane transporter activity” (GO:0015078). Prevailing terms of each GO category are summarized in Additional file 11: Figure S4. Among annotated proteins, non of transcription factor families related to the known ligule genes (including MADS box, MYB, and SBP) were found. Nevertheless,

## Discussion

Phenotypic characterization the *Ae. tauschii* Liguleless mutant revealed that the mutation affects the ligular region formation, but reproductive development is normal. In addition to defects in the ligule region (the absence of a ligule and auricles), an obvious characteristic feature of the LM leaf phenotype is a shortened leaf blade according to our study. We also found that plant height, the number of tillers, spike length, and the number of spikelets per spike in the NIL-*Lg*^*t*^ line are somewhat less than those in its SIB-line, but the difference is significant only in spike length. Wheat and rye liguleless forms do not show a similar reduction in the above-mentioned quantitative parameters of phenotypic traits [5, 6, 38]. Nevertheless, maize semidominant mutant *Lgn-R* possesses narrower and shorter leaves with a defective ligule and auricle. Leaf blade morphology in *Lgn-R* was found to be affected moderately*.* Reproductive development is abnormal in the *Lgn-R* mutant [39].

Busch et al. [40] proposed a conserved genetic system that establishes axillary meristems and determines leaf shape. In barley, recessive mutations in gene *ELIGULUM-A* (*ELI-A*) produce dwarfed plants with fewer tillers and disrupt the leaf blade–sheath boundary, thereby producing liguleless leaves [41, 42]. *ELI-A* is predicted to encode an unannotated protein containing an RNase H–like domain that contributes to leaf and lateral branch development [42].

Recessive mutations in the barley *Uniculme4* (*Cul4*) gene, which codes a BROAD-COMPLEX, TRAMTRACK, BRIC-À-BRAC-ankyrin protein closely related to *Arabidopsis thaliana* BLADE-ON-PETIOLE1 (BOP1) and BOP2, cause reduced tillering and alterations in leaf proximal–distal patterning. Loss of *Cul4* results in a liguleless phenotype and reduced tillering, indicating a conserved role of this protein in lateral organ initiation and proximal–distal patterning [43]. Although we found a slightly reduced number of tillers in NIL-*Lg*^*t*^ compared to its SIB-line, this difference is not significant and *Lg*^*t*^ is likely to contribute only to leaf development and does not participate in axillary-bud formation and reproductive development.

In Triticeae species, liguleless phenotypes are usually inherited as recessive traits. It has been found that liguleless phenotypes of wheat [23], rye [15, 16], and barley [17] are under the control of recessive mutation in genes located on group-2 chromosomes. Wheat genes *lg1*, *lg2*, and *lg3* [18–23], rye *el* [15, 16], and barley *li* [44] are located on the long arm of group 2 chromosomes, whereas barley gene *eli-A* has been mapped to chromosome 2HS.

Recently, the *Lg*^*t*^ mutant gene for the liguleless phenotype of *Ae. tauschii* was mapped to chromosome 2DL, taking into account its linkage to the *Xbarc159* SSR marker [26]. Nonetheless, on the reported map, the *Xbarc159* marker is not linked to any other 2DL markers [26]. Another inconsistency is that the *Lg*^*t*^ gene is no linked to the *Iw2* gene located on chromosome 2DS [26]*.*

Here, we applied the DArTseq approach to localize the *Lg*^t^ gene on highly saturated *Ae. tauschii* genetic maps. Given the complexity of mapping of this gene in a previous work [26], we chose only highly informative codominant DArTseq-derived SNP markers to construct *Ae. tauschii* molecular-genetic maps. Thus, the *Lg*^*t*^ (*qLg*^*t*^) gene was mapped to chromosome 5DS, and its location was confirmed by the results of SSR- and SNP-genotyping of NIL-*Lg*^*t*^ and its SIB-line. We did not find any additional QTLs for the trait, including those on chromosome 2D, and *qLg*^*t*^ (5DS) was the only identified QTL. This means that the liguleless trait of LM is inherited as a Mendelian trait, but some difficulties with the mapping of *Lg*^*t*^ as a single gene might be a result of its incomplete penetrance.

The *Ae. tauschii* genome sequence was published very recently [33], and this achievement enables identifying positions of DNA markers on *Ae. tauschii* pseudomolecules and delimiting the genome region harboring a gene of interest. The sequences of markers flanking the *Lg*^*t*^ gene (3960013_002 and 1077530_0065) were aligned to the *Ae. tauschii* genome sequence. Genes within this interval were identified as candidate genes on the basis of the position of the flanking markers. Although the list of candidate genes should be shortened by further experimental work (e.g., construction of new recombinants), the obtained information allowed us to rule out *Ae. tauschii* orthologs of known cereal genes for the liguleless trait as candidates for *Lg*^*t*^*.* Of these, sequences homologous to genes *lg1* (chr2, AET2Gv21102800, XM_020336542.1), *lg2* (chr3, AET3Gv20832700, XM_020334604.1), *Lg4* (chr1, AET1Gv20185800, XM_020322669.1), *Knox1* (chr4, AET4Gv20120200, XM_020291993.1), and *Lgn1* (chr7, AET7Gv20569100, XM_020291616.1) have a chromosomal location different from that of *Lg*^*t*^ (5DS), and sequence(s) homologous to *LG3* have not been found in either the *Ae. tauschii* genome (http://aegilops.wheat.ucdavis.edu/jbrowse/index.html?data) or bread wheat genome (https://urgi.versailles.inra.fr/blast/). Barley genes *Cul4* and *ELI-A*, whose recessive mutations cause liguleless phenotypes, were mapped to nonsynthenic chromosomes 3HL [43] and 2HS [42], respectively. The *Lg*^*t*^ gene is located in the region of chromosome 5DS that shares synteny with bread wheat 5DS, rice Os12, sorghum Sb2, and Brachypodium Bd4 chromosomes [33, 34].

## Conclusions

In the present study, we characterized the *Ae. tauschii* liguleless induced mutant, whose phenotype is under the control of the *Lg*^*t*^ dominant mutation. The dominant mode of inheritance of the liguleless trait in Triticeae species is reported for the first time. Morphological features of LM suggest that *Lg*^*t*^ is involved in the control of leaf development, mainly in leaf proximal–distal patterning. The gene was genetically mapped to chromosome 5DS by means of DArTseq-derived SNP markers. *Lg*^*t*^ is located in a region of conserved synteny with bread wheat 5DS, rice Os12, sorghum Sb2, and Brachypodium Bd4 chromosomes. A list of identified candidate genes for *Lg*^*t*^ does not contain *Ae. tauschii* orthologs of any well-characterized cereal genes whose mutations cause liguleless phenotypes. The characterized *Lg*^*t*^ mutant represents a new model for further research into plant leaf patterning and differentiation.

## Additional files

Additional file 1: **Table S4.** Primers for sequencing the regions, containing DArTseq-derived SNPs. (XLSX 343 kb)

Additional file 2: **Table S6.** SSR-analysis of the *Lg*^t^-locus in Liguleless Mutant and its SIB-line. (XLSX 10 kb)

Additional file 3: **Table S1.** List of DArTseq-derived SNPs. (DOCX 145 kb)

Additional file 4: **Table S2.** Marker information for molecular-genetic maps of *Ae. tauschii*. (XLSX 33 kb)

Additional file 5: **Figure S1.** Molecular-genetic maps of *Ae. tauschii* chromosomes. (PPTX 46 kb)

Additional file 6: **Table S3.** Positions of markers on *Ae. tauschii* molecular-genetic maps. (DOCX 109 kb)

Additional file 7: **Figure S2.** Partial SNP and microsatellite maps of chromosome 2D, indicating position of the *Xgwm301* marker. (XLSX 13 kb)

Additional file 8: **Figure S3.** Genetic mapping of the gene that determines the liguleless trait of the *Ae. tauschii* Liguleless Mutant as a single Mendelian gene, *Lg*^t^, and a QTL, *qLg*^t^. (XLSX 11 kb)

Additional file 9: **Table S5.** SNP polymorphisms detected in NIL-*Lg*^t^ and its SIB-Line. (XLSX 11 kb)

Additional file 10: **Table S7.** Genes identified by similarity search and by ab initio prediction. (XLSX 38 kb)

Additional file 11: **Figure S4.** Pie charts of prevalent terms of three aspects of gene ontology. (DOCX 303 kb)
