# Supplementary material for: Characterization of a dominant mutation for the liguleless trait: Aegilops tauschii liguleless (Lgt)
Source: BMC Plant Biol. 2019 Feb 15;19(Suppl 1):55. doi: 10.1186/s12870-019-1635-z (PMC6393956; doi:10.1186/s12870-019-1635-z)
Supplement: Supplementary file 3 — Table S1. List of DArTseq-derived SNPs. (DOCX 145 kb) [file 12870_2019_1635_MOESM3_ESM.docx]

FigureS1. Molecular-genetic maps of *Ae. tauschii* chromosomes*.* Genetic distances indicated on the left side of linkage group in centiMorgans (cM), the marker names shown on the right side.

Marker name

Genetic distance (cM)

**1D**

**2D**

Genetic distance (cM)

Marker name

**3D**

Genetic distance (cM)

Marker name

Genetic distance (cM)

Marker name

**4D**

**5D**

Genetic distance (cM)

Marker name

Genetic distance (cM)

Marker name

**6D**

Genetic distance (cM)

Marker name

**7D**
