# Supplementary material for: Characterization of a dominant mutation for the liguleless trait: Aegilops tauschii liguleless (Lgt)
Source: BMC Plant Biol. 2019 Feb 15;19(Suppl 1):55. doi: 10.1186/s12870-019-1635-z (PMC6393956; doi:10.1186/s12870-019-1635-z)
Supplement: Supplementary file 5 — Figure S1. Molecular-genetic maps of Ae. tauschii chromosomes. (PPTX 46 kb) [file 12870_2019_1635_MOESM5_ESM.pptx]

## Slide 1
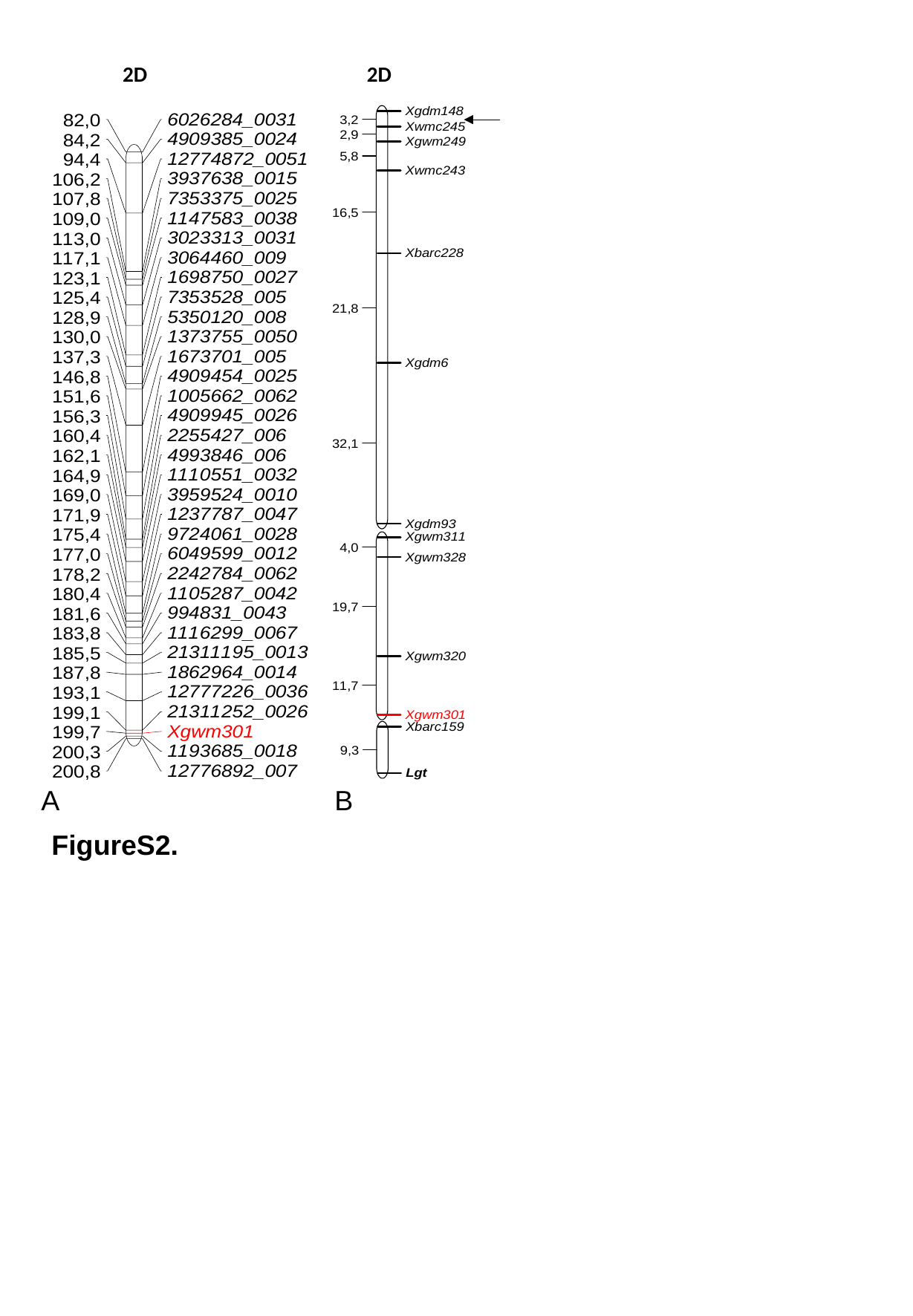

2D
2D
A
B
FigureS2.

## Slide 2
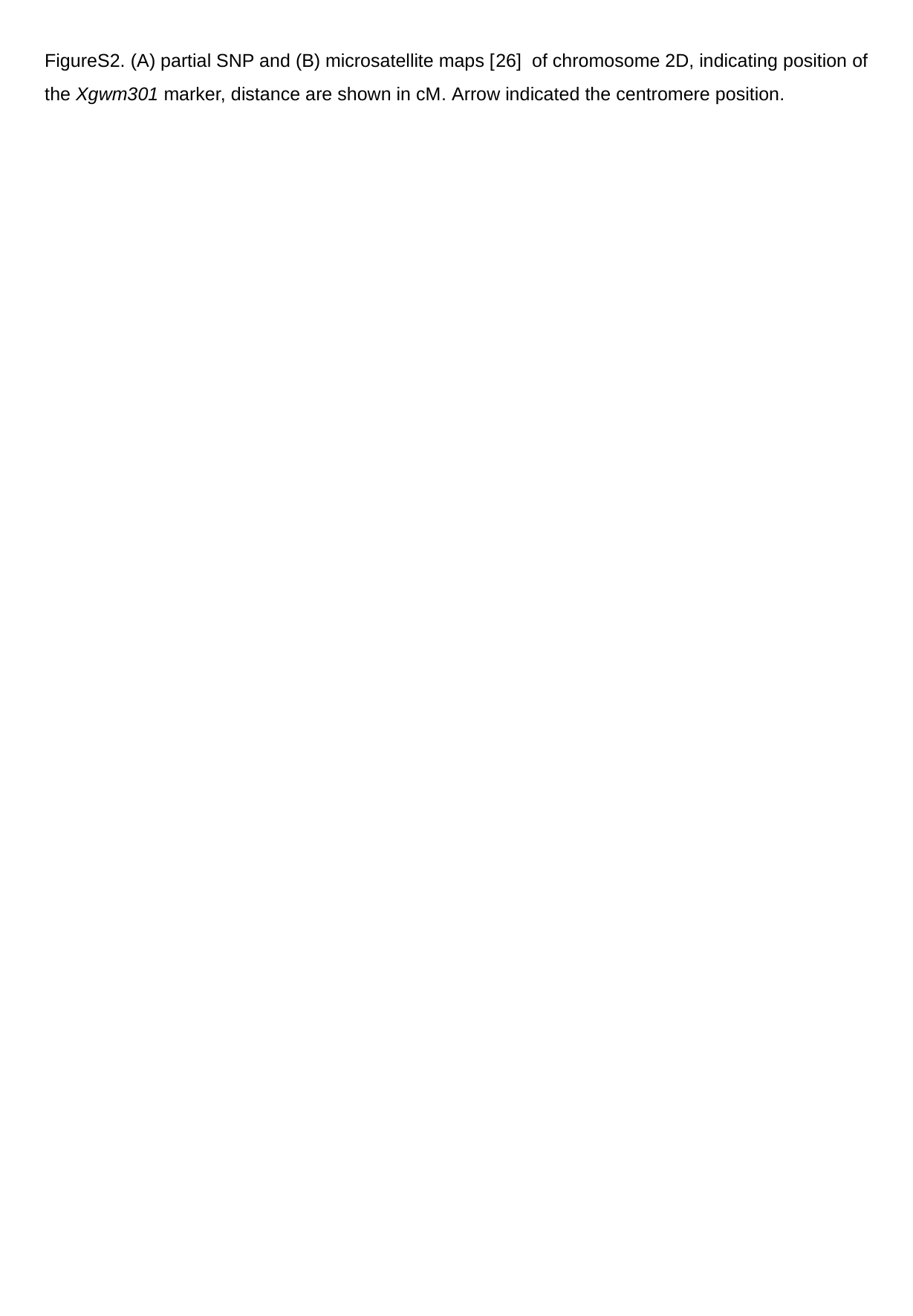

FigureS2. (A) partial SNP and (B) microsatellite maps [26] of chromosome 2D, indicating position of the Xgwm301 marker, distance are shown in cM. Arrow indicated the centromere position.
