# Supplementary material for: Characterization of a dominant mutation for the liguleless trait: Aegilops tauschii liguleless (Lgt)
Source: BMC Plant Biol. 2019 Feb 15;19(Suppl 1):55. doi: 10.1186/s12870-019-1635-z (PMC6393956; doi:10.1186/s12870-019-1635-z)
Supplement: Supplementary file 6 — Table S3. Positions of markers on Ae. tauschii molecular-genetic maps. (DOCX 109 kb) [file 12870_2019_1635_MOESM6_ESM.docx]

**5D**

**5D**

B

A

FigureS3. Genetic mapping of the *Lg^t^* gene that determines the liguleless trait of the *Ae. tauschii* Liguleless Mutant as a single Mendelian gene, *Lg^t^* (A) and a QTL, *qLg^t^* (B). Genetic distances in centimorgans (cM) are indicated on the left side of each linkage group, the marker names shown on the right side.
