# Supplementary material for: Characterization of a dominant mutation for the liguleless trait: Aegilops tauschii liguleless (Lgt)
Source: BMC Plant Biol. 2019 Feb 15;19(Suppl 1):55. doi: 10.1186/s12870-019-1635-z (PMC6393956; doi:10.1186/s12870-019-1635-z)
Supplement: Supplementary file 11 — Figure S4. Pie charts of prevalent terms of three aspects of gene ontology. (DOCX 303 kb) [file 12870_2019_1635_MOESM11_ESM.docx]

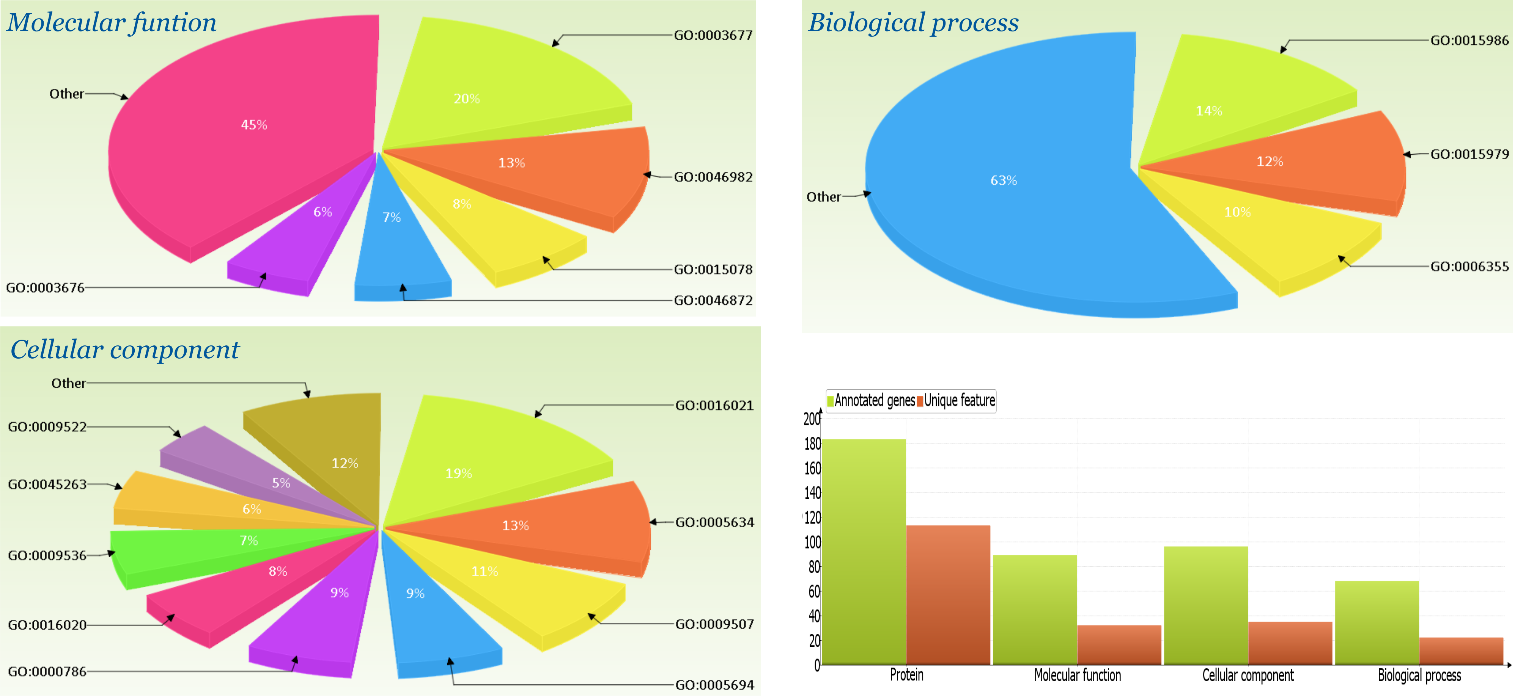


FigureS4. Pie charts of prevalent terms (more than 5% of all genes) of three aspects of gene ontology. The name and fraction of prevailing terms are indicated. Histogram represents the distribution of the number of annotated genes and unique terms for each GO aspect.
